# Memantine and the Kynurenine Pathway in the Brain: Selective Targeting of Kynurenic Acid in the Rat Cerebral Cortex

**DOI:** 10.3390/cells13171424

**Published:** 2024-08-26

**Authors:** Renata Kloc, Ewa M. Urbanska

**Affiliations:** Chair and Department of Experimental and Clinical Pharmacology, Medical University of Lublin, 20-090 Lublin, Poland; renata.kloc@umlub.pl

**Keywords:** memantine, neurodegeneration, kynurenine pathway, kynurenic acid, cortex

## Abstract

Cytoprotective and neurotoxic kynurenines formed along the kynurenine pathway (KP) were identified as possible therapeutic targets in various neuropsychiatric conditions. Memantine, an adamantane derivative modulating dopamine-, noradrenaline-, serotonin-, and glutamate-mediated neurotransmission is currently considered for therapy in dementia, psychiatric disorders, migraines, or ischemia. Previous studies have revealed that memantine potently stimulates the synthesis of neuroprotective kynurenic acid (KYNA) in vitro via a protein kinase A-dependent mechanism. Here, the effects of acute and prolonged administration of memantine on brain kynurenines and the functional changes in the cerebral KP were assessed in rats using chromatographic and enzymatic methods. Five-day but not single treatment with memantine selectively activated the cortical KP towards neuroprotective KYNA. KYNA increases were accompanied by a moderate decrease in cortical tryptophan (TRP) and L-kynurenine (L-KYN) concentrations without changes in 3-hydroxykynurenine (3-HK) levels. Enzymatic studies revealed that the activity of cortical KYNA biosynthetic enzymes ex vivo was stimulated after prolonged administration of memantine. As memantine does not directly stimulate the activity of KATs’ proteins, the higher activity of KATs most probably results from the increased expression of the respective genes. Noteworthy, the concentrations of KYNA, 3-HK, TRP, and L-KYN in the striatum, hippocampus, and cerebellum were not affected. Selective cortical increase in KYNA seems to represent one of the mechanisms underlying the clinical efficacy of memantine. It is tempting to hypothesize that a combination of memantine and drugs could strongly boost cortical KYNA and provide a more effective option for treating cortical pathologies at early stages. Further studies should evaluate this issue in experimental animal models and under clinical scenarios.

## 1. Introduction

A total of 95–99% of ingested tryptophan (TRP) enters the kynurenine pathway (KP), while the remaining pool of this amino acid is used in the synthesis of serotonin, melatonin, and proteins [1]. Kynurenines, downstream metabolites of TRP formed in the periphery and brain, are known to play vital roles in various physiological processes, including modulation of the immune response and cellular survival [2]. Compounds synthesized along the KP possess pleiotropic activity. Potentially harmful, cytotoxic molecules include primarily 3-hydroxykynurenine (3-HK), 3-hydroxyanthranilic acid (3-HANA), and quinolinate (QUIN). QUIN-induced neurodegeneration results from excessive stimulation of the N-methyl-D-aspartate (NMDA) receptors at the agonist-binding site, whereas the neurotoxicity of 3-HK and 3-HANA is linked largely with the generation of free radicals [3]. 

The major cytoprotective molecule in the KP is kynurenic acid (KYNA). KYNA binds with the highest affinity to the strychnine-insensitive glycine site at the NR1 subunit of the NMDA receptor and with a lower affinity to α-amino-3-hydroxy-5-methyl-4-isoxazolopropionic acid (AMPA) and kainate receptors [4]. It is generally accepted that KYNA acts as a noncompetitive antagonist of nicotinic α7 receptors, although some findings do not corroborate this effect [5,6]. KYNA also stimulates the aryl hydrocarbon receptor (AHR), a transcription factor involved in xenobiotic metabolism [7], and acts as an agonist of the orphan G protein-coupled receptor G35 (GPR35) expressed on the surface of immunocompetent cells and glia. Through GPR35, KYNA may modulate the immune response and glial metabolism, possibly due to decreased cAMP formation [4,8]. Furthermore, the anti-inflammatory and antioxidant effects of KYNA have been shown in various paradigms [9,10]. In the brain, KYNA is mostly of astrocytic origin [11]. Its formation depends primarily on the enzymatic conversion of its precursor, L-KYN, which easily penetrates through the blood–brain barrier from the periphery. Kynurenine aminotransferases (KATs) I and II differ according to their activity, subcellular localization, optimal pH, and affinity for co-factors [12]. 

Numerous experimental and human studies on neurological and psychiatric disorders have revealed disturbed metabolism of TRP along the KP [3]. Hence, cytoprotective and neurotoxic kynurenines were identified as possible therapeutic targets in brain disorders including Alzheimer’s disease (AD) [13,14]. Peripheral activation of the KP, accompanied by decreased KYNA and increased 3-HK and QUIN formation, were detected in AD. Therefore, the relative overabundance of neurotoxic metabolites in the course of the disease has been suggested as one of its causative factors [8,15]. Others, however, have showed activation of the KP and increased serum KYNA levels [16]. The status of central KP in AD is also not fully clarified. Most of the data show a decline in central KYNA and increased 3-HK and QUIN both in experimental models and among patients with dementia [17]. Nevertheless, activation of the KP with an increase in KYNA and QUIN in the CSF of AD patients has also been shown [18]. Functional studies revealed an increased ratio of 3-HK/KYN, which correlated with other AD biomarkers in CSF [19]. A recent meta-analysis showed a central increase in the KYNA/L-KYN ratio and lower serum KYNA in AD [20].

Memantine is a non-competitive NMDA receptor antagonist with a moderate affinity, blocking the ion channel in a membrane potential-dependent manner [21]. It is characterized by rapid kinetics of binding and dissociation from the receptor and therefore does not interfere with physiological activation of the NMDA receptor [22]. Importantly, memantine prevents excitotoxicity in various models but does not attenuate learning and memory processes [23]. Memantine impairs β-amyloid protein-induced toxicity and related signaling processes [23]. The neuroprotective effects of the drug were also linked with an enhanced expression of neurotrophic factors, inhibition of excessive microglia activation, and a reduction in the release of pro-inflammatory factors such as free radicals, nitric oxide, or tumor necrosis factor-α (TNF-α) [24,25]. The favorable profile of memantine in the treatment of Alzheimer’s disease (AD) dementia has been confirmed by many randomized controlled clinical trials. The drug was shown to exert multiple beneficial effects on cognitive function, including an improvement in behavioral deficits, daily performance, and overall patient functioning [26,27]. 

Previous research has revealed that memantine potently stimulates KYNA synthesis in rat brain cortical slices and mixed primary glial cultures in a protein kinase A (PKA)-dependent mechanism [28]. This study aimed to evaluate the functional alterations in the central KP after acute and prolonged administration of memantine. The levels of TRP and its metabolites were studied in four brain regions including the cerebral cortex, hippocampus, striatum, and cerebellum. Furthermore, analyses of the ratios between different kynurenines were performed and served as indicators of the KP activity towards either neuroprotective or neurotoxic metabolites.

## 2. Materials and Methods

### 2.1. Experimental Protocol

The experiments were performed on male adult Wistar rats with an initial body mass of 200–220 g. The animals were housed under standard laboratory conditions, with a 12 h light/dark cycle, 18 °C environmental temperature, and free access to food and water. The animals were randomly assigned to the experimental groups (n = 10). The control animals received intraperitoneal injections of physiological saline once or for 5 days. Memantine was administered intraperitoneally (i.p.) at a dose of 20 mg/kg b.w. acutely, or for 5 days. The dose was chosen based on the available literature and was comparable to the serum levels achieved in patients treated with memantine under clinical scenarios [29,30,31]. To minimize the number of used animals, a single dose of memantine was used in the experimental protocol. Injections were always carried out on the same day time. The animals were decapitated one hour after the administration of the drug or saline. After decapitation, the brains were rapidly removed from the skull and the cortices, hippocampi, striata, and cerebella were quickly dissected. The brain structures were stored at −72 °C until further studies were performed. The experiments were carried out in agreement with the European Council Directive for the use of animals in experimental research and with permission obtained from the local Ethical Committee in Lublin, nr 45/2019.

### 2.2. Brain Levels of Kynurenines

On the day of analysis, the tissues were weighed and pulse-homogenized at a ratio of 1:10 (weight/volume) in distilled water using an ultrasonic homogenizer (Bandelin Sonopuls, Berlin, Germany). During homogenization, the tubes that contained the samples were submersed in a water bath (4 °C). The homogenate was centrifuged (12,000 rpm, 10 min, 4 °C), and the resulting supernatant was transferred to Eppendorf tubes and deproteinized using 8% HClO4 (100 µL per 500 µL of supernatant). The whole mixture was mixed and centrifuged again (12,000 rpm, 10 min, 4 °C). The supernatant was stored at −72 °C until further analyses.

### 2.3. Enzymatic Analyses of Semi-Purified Kynurenine Aminotransferases (KAT I and II)

The analyses of KAT I and KAT II activity were carried out according to the method described by Guidetti et al. [32], with modifications [33]. The cerebral cortices were weighed and homogenized at a ratio of 1:10 (weight/volume) in dialysis buffer (5 mM Tris-acetate, 2 mM pyridoxal-5-phosphate, 10 mM 2-mercapto-ethanol) at 4 °C. The homogenate was centrifuged (12,000 rpm, 10 min, 4 °C). The supernatant was placed in cellulose dialysis membranes (Dialysis tubing, Sigma-Aldrich, St. Louis, MO, USA) and dialyzed for 12–14 h against dialysis buffer at 4 °C. The incubation mixture contained 100 µL of dialysate and 100 µL of the respective buffer (for KAT I or KAT II). The KAT I buffer was composed of the following (final concentrations): 2 µM L-KYN, 70 µM 5-pyridoxal phosphate, 1 mM pyruvic acid, 150 mM Tris-acetate buffer pH 9.5. The KAT II contained 2 µM L-KYN, 70 µM 5-pyridoxal phosphate, 1 mM pyruvic acid, 150 mM Tris-acetate buffer, pH 7.4, and 2 mM L-glutamine (KAT I inhibitor). Blank samples were obtained via heat inactivation of the dialysate performed for 15 min at 95 °C. The semi-purified enzymatic preparations were incubated at 37 °C for 2 h. At the end of the incubation time, the samples were immediately cooled to 4 °C, acidified with 1 mL of 0.1 N HCl and 14 µL of 50% TCA, mixed, and centrifuged (12,000 rpm, 10 min, 4 °C). The supernatants were used for further quantification of the KYNA contents. 

### 2.4. Quantification of Tryptophan, L-Kynurenine, and Kynurenic Acid in Brain Samples

Quantitative determinations of TRP, L-KYN, and KYNA contents in the brain samples were performed using an ultra-high-pressure liquid chromatograph (UHPLC) with a fluorescence detector (UltiMate 3000 Analytical systems, Thermo Fisher Scientific, Waltham, MA, USA) according to the method described by Zhao [34]. A 250 × 4.6 mm analytical column with a pore size of 5 μm (Agilent HC-C18) was used for the determination. The mobile phase (3 mM zinc acetate, 20 mM sodium acetate, and 7% acetonitrile, pH 6.2) flow rate was 1 mL/min. Analyses were carried out simultaneously using fluorescence detection for KYNA (excitation wavelength of 344 nm and emission wavelength of 398 nm) and UV detection for L-KYN and TRP (365 nm and 250 nm wavelength, respectively). Calibration curves were generated for each substance and covered the range of 0.1–1.0 pmol/100 μL for KYNA, 10.0–200.0 pmol/100 μL for L-KYN, and 100.0–2000.0 pmol/100 μL for TRP. Chromeleon 7.2 software was used to control the HPLC systems and record the chromatographic data.

### 2.5. Quantification of 3-Hydroxykynurenine in Brain Samples

The 3-HK content in the samples was assessed using an HPLC system with an electrochemical detector (Coulochem III, ESA, Paris, France) connected to an analytical cell with the oxidation voltage set at +0.20 V according to the method described by Heyes and Quearry [35]. An ESA catecholamine HR-80, 3 µm, reversed-phase C18 column was perfused with a mobile phase consisting of 2% acetonitrile, 0.9% triethylamine, 0.59% phosphoric acid, 0.27 mM sodium EDTA and 8.9 mM heptanesulfonic acid (flow rate 0.3 mL/min). Chromeleon 7.2 software was used to control the HPLC systems and record chromatographic data.

### 2.6. Statistical Analyses

Data were calculated as mean values ± S.D. and are shown as a percentage of the mean control values, which were set at 100%. Ratios were calculated separately for each animal, and an average mean ratio ± S.D. was subsequently drawn. Initial analyses of the normal distribution of the data were performed first, followed by either the Student’s *t*-test for Gaussian-distributed data or the Mann–Whitney U test. The statistical significance of the results was set at *p* < 0.05.

## 3. Results

### 3.1. The Influence of Acute and 5-Day Administration of Memantine on the Content of Kynurenic Acid, Kynurenine, Tryptophan, and 3-HK in the Cortex

**Figure 1:**
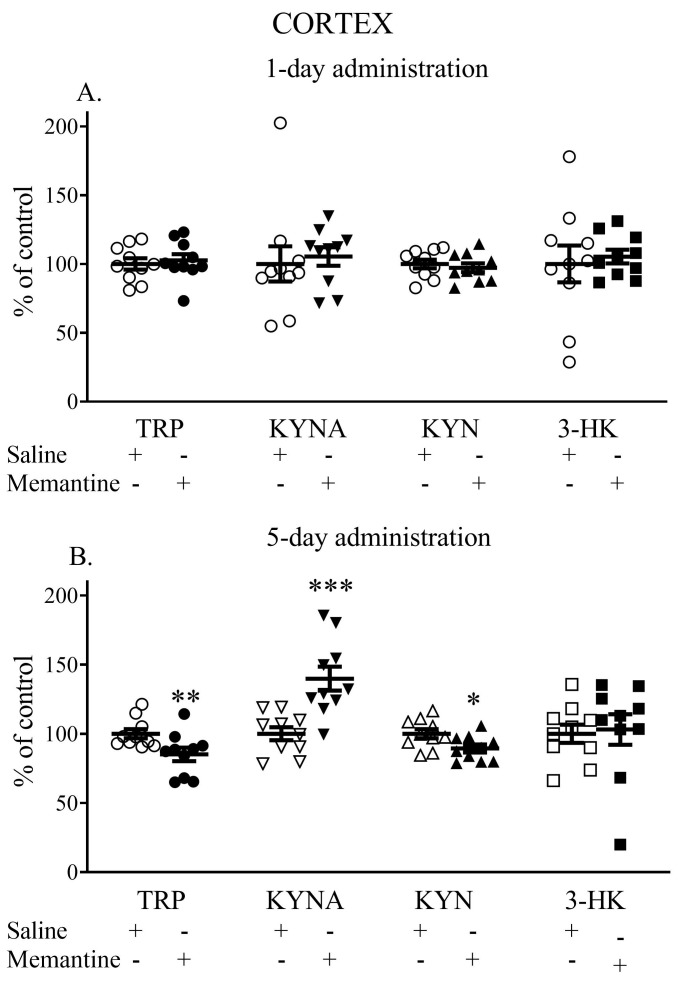
The effects of an acute (**A**) and 5-day treatment (**B**) with memantine on the levels of selected kynurenines in the cerebral cortex. TRP—tryptophan, KYNA—kynurenic acid, L-KYN—L-kynurenine, 3-HK—3-hydroxykynurenine. Data are shown as the percentage of mean control values set at 100%. Statistical significance * *p* < 0.05; ** *p* < 0.01; *** *p* < 0.001 vs. control (receiving saline injection).

Acute injection of memantine did not change the levels of TRP, KYNA, KYN, and 3-HK in the cerebral cortex. The TRP content in the cerebral cortex was 118.6 ± 19.9 vs. 122.6 ± 11.7 (nmol/g tissue), the KYNA content was 146.06 ± 30.43 vs. 131.64 ± 41.25 (pmol/g tissue), the L-KYN content (nmol/g tissue) reached in the cerebral cortex was 4.29 ± 5.49 vs. 4.52 ± 4, and the 3-HK content (pmol/g tissue) was 230.43 ± 41.22 vs. 218.75 ± 84.97 (Figure 1A).

A 5-day treatment with memantine decreased the content of TRP in the cerebral cortex (86% of control, *p* < 0.01 vs. control) (89.82 ± 22.52 vs. 107.86 ± 27.2 nmol/g tissue), significantly increased cortical KYNA levels (139% of the control; *p* < 0.001) (176.23 ± 41.73 vs. 127.19 ± 23.05 pmol/g tissue), decreased cortical L-KYN levels (90% of control; *p* < 0.05) (4.33 ± 0.44 vs. 4.79 ± 0.407 nmol/g tissue), and did not affect the levels of 3-HK (222.95 ± 50.03 vs. 216.30 ± 41.64 pmol/g tissue) (Figure 1B).

### 3.2. The Influence of Acute and 5-Day Administration of Memantine on the Content of Kynurenic Acid, Kynurenine, Tryptophan, and 3-HK in the Hippocampus

**Figure 2:**
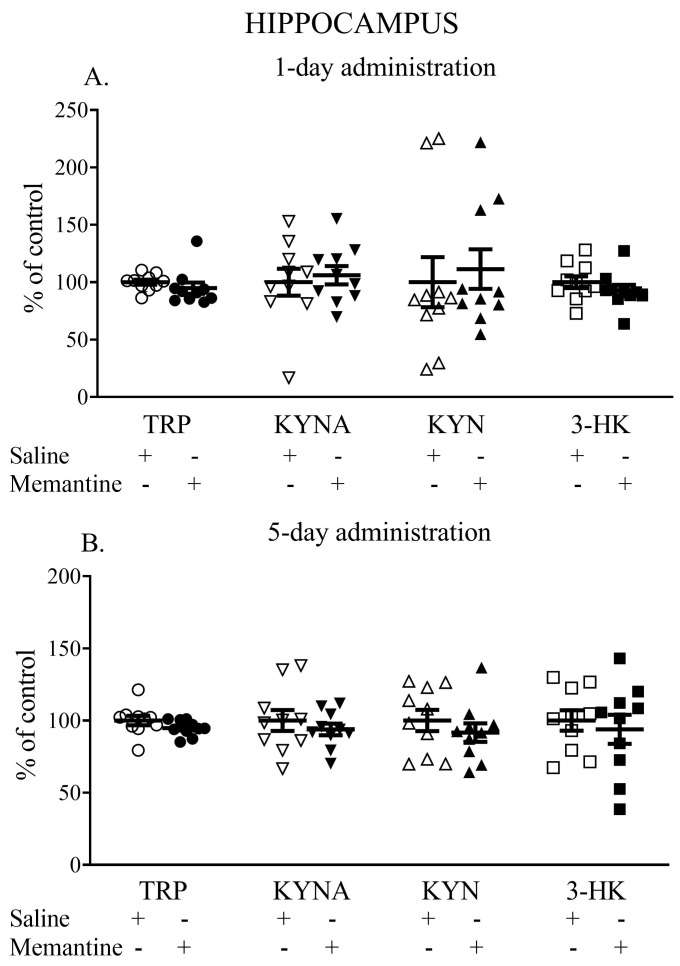
The effects of acute (**A**) and a 5-day treatment (**B**) with memantine on the levels of selected kynurenines in the hippocampus of rats. Data are shown as the percentage of mean control values set as 100%. TRP—tryptophan, KYNA—kynurenic acid, L-KYN—L-kynurenine, 3-HK—3-hydroxykynurenine.

Memantine given acutely did not influence the hippocampal TRP, KYNA, KYN, or 3-HK levels. The TRP content (nmol/g tissue) was 183.16 ± 18.00 vs. 187.8 ± 13.3, the KYNA content (pmol/g tissue) was 50.46 ± 12.10 vs. 52.46 ± 11.42, the L-KYN content (nmol/g tissue) was 2.44 ± 1.2 vs. 2.5 ± 1.36, and the 3-HK content (pmol/g tissue) was 5.66 ± 0.96 vs. 6.10 ± 0.95 (Figure 2A).

Similarly, a 5-day administration of memantine did not influence TRP, KYNA, KYN, or 3-HK levels. The TRP content (nmol/g tissue) reached 153.55 ± 8.85 vs. 162.04 ± 16.79, the KYNA content (pmol/g tissue) was 176.23 ± 41.73 vs. 127.19 ± 23.05, the L-KYN content (nmol/g tissue) was 2.68 ± 0.47 vs. 2.74 ± 0.63, and the 3-HK content (pmol/g tissue) was 4.83 ± 1.61 vs. 5.14 ± 1.15 (Figure 2B). 

### 3.3. The Influence of Acute and 5-Day Administration of Memantine on the Content of Kynurenic Acid, Kynurenine, Tryptophan, and 3-HK in the Striatum

**Figure 3:**
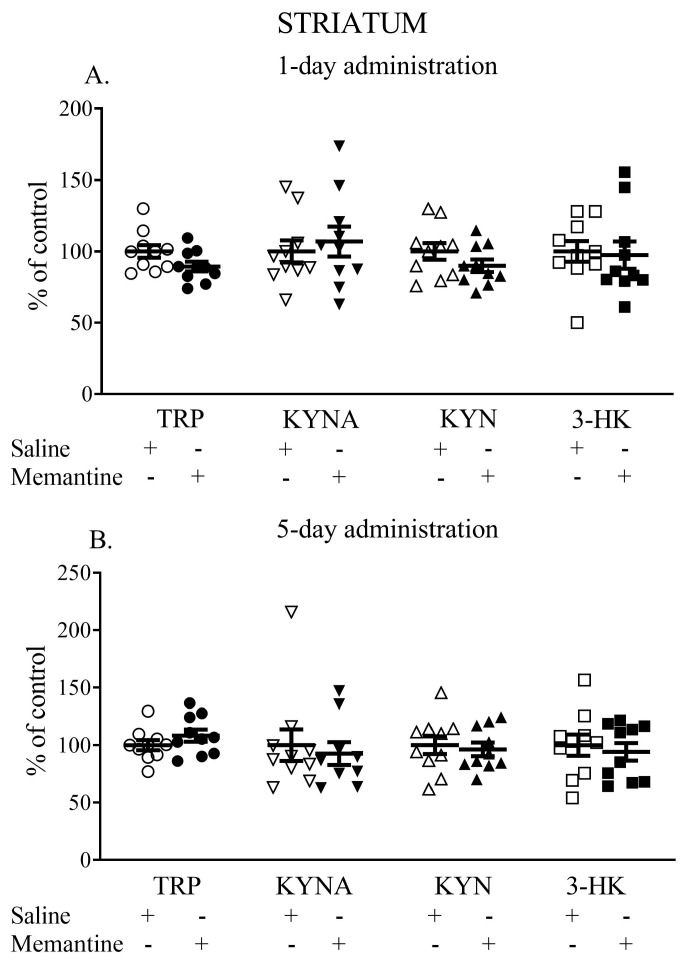
The effects of acute (**A**) and a 5-day treatment (**B**) with memantine on the levels of selected kynurenines in the striatum of rats. Data are shown as the percentage of mean control values set as 100%. TRP—tryptophan, KYNA—kynurenic acid, L-KYN—L-kynurenine, 3-HK—3-hydroxykynurenine.

Single administration of memantine did not affect the striatal TRP, KYNA, KYN, or 3-HK levels. The TRP content (nmol/g tissue) was 9.1 ± 0.9 vs. 9.7 ± 0.8, the KYNA content (pmol/g tissue) was 70.73 ± 17.29 vs. 68.26 ± 22.16, the L-KYN content (nmol/g tissue) was 3.44 ± 1.01 vs. 3.52 ± 0.58, and the 3-HK content (pmol/g tissue) was 663.13 ± 218.92 vs. 680.72 ± 165.13 (Figure 3A). 

Similarly, memantine administered for 5 days did not change the levels of the studied metabolites. The TRP content (nmol/g tissue) was 10.2 ± 1.39 vs. 9.74 ± 1.34, the KYNA content (pmol/g tissue) was 54.63 ± 14.80 vs. 54.75 ± 10.68, the L-KYN content (nmol/g tissue) was 2.77 ± 0.54 vs. 2.87 ± 0.7, and the 3-HK content (pmol/g tissue) was 646.01 ± 168.92 vs. 655.88 ± 158.23 (Figure 3B).

### 3.4. The Influence of Acute and 5-Day Administration of Memantine on the Content of Kynurenic Acid, Kynurenine, Tryptophan, and 3-HK in the Cerebellum

**Figure 4:**
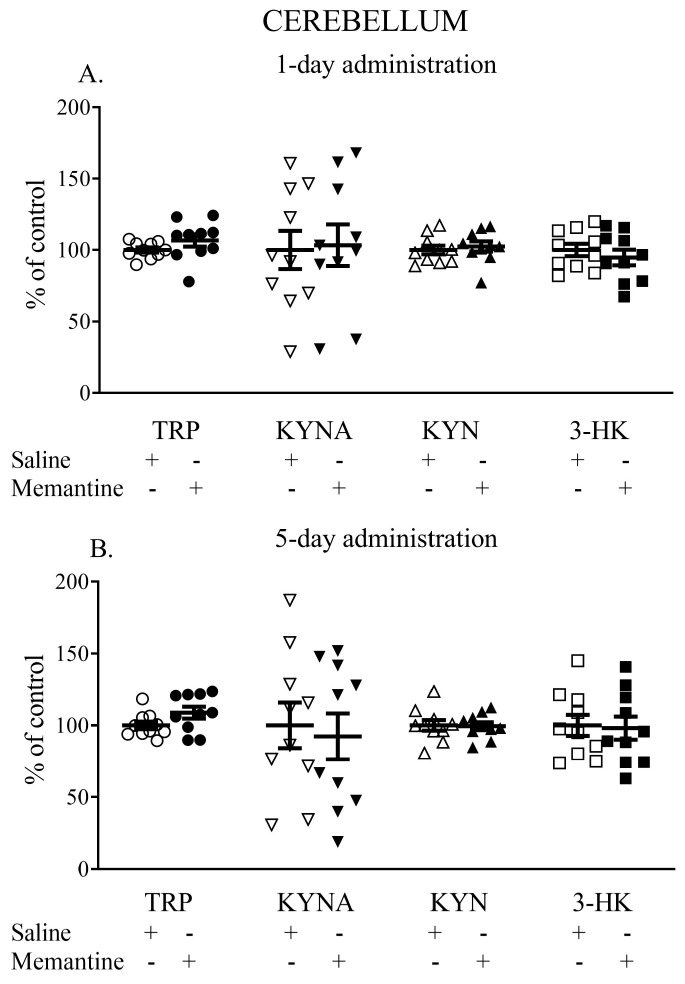
The effects of acute (**A**) and a 5-day treatment (**B**) with memantine on the levels of selected kynurenines in the cerebellum of rats. Data are shown as the percentage of mean control values set as 100%. TRP—tryptophan, KYNA—kynurenic acid, L-KYN—L-kynurenine, 3-HK—3-hydroxykynurenine.

One-day administration of memantine did not influence the cerebellar TRP, KYNA, KYN, or 3-HK levels. The TRP content (nmol/g tissue) was 15.03 ± 1.2 vs. 14.5 ± 0.87, the KYNA content (pmol/g tissue) was 8.70 ± 3.17 vs. 8.5 ± 2.70, the L-KYN content (nmol/g tissue) was 5.31 ± 0.61 vs. 5.12 ± 0.7, and the 3-HK content (pmol/g tissue) was 16.41 ± 2.14 vs. 17.32 ± 2.36 (Figure 4A). 

Similarly, a 5-day administration of memantine did not affect the levels of the studied metabolites. The TRP content (nmol/g tissue) was 17.99 ± 2.42 vs. 17.86 ± 1.88, the KYNA content (pmol/g tissue) was 7.89 ± 3.36 vs. 7.70 ± 3.17, the L-KYN content (nmol/g tissue) was 4.69 ± 0.43 vs. 4.72 ± 0.58, and 3-HK content reached 15.62 ± 4.05 vs. 15.93 ± 3.69 (Figure 4B).

### 3.5. The Effects of Acute and 5-Day Administration of Memantine on the Cortical Activity of Semi-Purified Kynurenine Aminotransferases I and II

**Figure 5:**
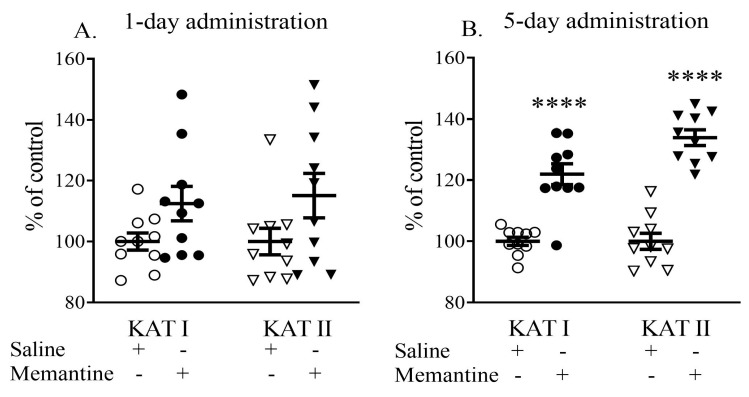
The effects of acute (**A**) or a 5-day (**B**) administration of memantine on the ex vivo activity of kynurenine aminotransferases (KATs) I and II. Data are shown as the percentage of mean control values set at 100%. Statistical significance **** *p* < 0.001 vs. control.

Single administration of memantine (20 mg/kg, i.p.) did not affect the activity of semi-purified cortical KAT I and KAT II. Memantine administered for 5 days significantly increased cortical KAT I and KAT II activity to 120% and 132% of the control values, respectively (both *p* < 0.001 vs. control) (Figure 5).

### 3.6. The Influence of Memantine on TRP/KYN, KYN/KYNA, KYN/3-HK, and KYNA/3-HK Ratios

A single administration of memantine did not affect the ratio TRP/L-KYN, TRP/KYNA, L-KYN/KYNA, 3-HK/KYNA, L-KYN/3-HK, or TRP/3-HK in the cerebral cortex, striatum, hippocampus, or cerebellum compared to their respective control groups (Table 1).

In contrast, memantine administered for 5 days reduced the ratio of TRP/KYNA, L-KYN/KYNA, and 3-HK/KYNA in the cerebral cortex (62%, 65%, and 72% of the control, respectively, *p* < 0.001 and *p* < 0.05 vs. control) and did not influence the ratios of TRP/L-KYN, L-KYN/3-HK, or TRP/3-HK (Table 2). Memantine did not affect the TRP/L-KYN, TRP/KYNA, L-KYN/KYNA, 3-HK/KYNA, or L-KYN/3-HK indices in the striatum, hippocampus, or cerebellum of the rats (Table 2). 

## 4. Discussion

The presented data indicate that prolonged but not acute peripheral administration of memantine selectively activates the cortical KP towards neuroprotective KYNA under in vivo conditions. These results corroborate our previous finding demonstrating that memantine enhances KYNA synthesis in cortical slices and mixed glial cultures in a protein kinase C-dependent way [28]. KYNA increases were accompanied by a moderate decrease in cortical TRP and L-KYN concentrations without change in 3-HK levels. Furthermore, TRP/KYNA, L-KYN/KYNA, and 3-HK/KYNA ratios were reduced by almost 30–40% in the cerebral cortex. 

The above effects can be interpreted in two ways: (1) as an increase in the activity of an enzyme that metabolizes TRP to KYNA precursor, L-KYN, or (2) as a result of increased utilization of TRP and L-KYN to produce KYNA with a steady supply of both precursors from the periphery to the CNS. The first effect seems much less likely, as an increase in the activity of the first, rate-limiting enzyme of the KP, indoleamine 2,3-dioxygenase (IDO), is usually associated with higher L-KYN concentrations and decreased TRP/L-KYN ratios, which was not observed here. A confirmation of the second hypothesis would be an increase in KAT I and KAT II activity in vivo, causing a depletion of the substrate pool. Consequently, higher KYNA production would be accompanied by a decrease in the concentrations of the immediate precursor L-KYN and the TRP preceding L-KYN along the KP, as indeed was the case here. Furthermore, enzymatic studies confirmed that the activity of KAT I and KAT II ex vivo was stimulated in animals exposed to the prolonged administration of memantine. Given that memantine does not directly influence the activity of KAT I and II [28], their higher activity may result from an increased expression of the respective genes. Our results are consistent with data showing that the expression of mRNA for KAT II in the rat cerebral cortex is stimulated by memantine (10 mg/kg) [36]. 

Of note, the concentrations of KYNA, 3-HK, TRP, and L-KYN in the striatum, hippocampus, and cerebellum were not affected by either single or 5-day treatment with memantine. Selective modulation of TRP metabolism in the kynurenine pathway has been described previously. The use of antidepressants in rats reproducibly increased KYNA content in the hippocampus, while changes in the cerebral cortex were observed after the administration of only two of the four drugs tested [37]. No changes in KYNA levels were observed in the striatum [37]. An enhanced mRNA expression of genes for KAT I and KAT II has also been shown after the administration of antidepressants and in rat primary glial cultures incubated for 24 to 48 h in the presence of antidepressants [38]. These data indicate the potential antidepressant activity of memantine, and indeed, such effects have been observed in animal models of depression [39]. Considering the above, it can be assumed that an increase in cortical KYNA levels is one of the mechanisms contributing to memantine’s antidepressant activity. Notably, randomized clinical trials indicate that memantine may be useful as an adjunctive drug in antidepressant therapy [39,40].

The previous results suggest that memantine increases central KYNA production through a mechanism mediated by PKA activity [28]. A PKA-dependent stimulation of KAT expression/activity leading to an increase in KYNA synthesis was also described in the presence of β-hydroxybutyrate, a ketone body [41]. Memantine-induced increases in cortical KYNA levels may reflect higher expression of KAT I and KAT II, mediated by a PKA-related mechanism. This hypothesis requires further studies.

Memantine, a structural derivative of amantadine, was initially described as an agent that interferes with the metabolism of neurotransmitters such as dopamine, noradrenaline, or serotonin and was proposed as a drug able to ameliorate the symptoms of Parkinson’s disease [42]. Following the discovery that memantine acts as an uncompetitive NMDA receptor antagonist, the drug was approved for the treatment of neurodegenerative disorders such as AD and other forms of dementia [43]. More than two decades of research expanded our knowledge about the drug, and currently, memantine is also considered a promising therapy in a variety of other brain pathologies, including bipolar disease, depression and schizophrenia, migraines, or neuropathic pain [44,45,46,47]. Memantine has also gained attention as a drug able to improve the outcomes of patients suffering from ischemic stroke [48].

The therapeutic efficacy of memantine is linked mostly with the blockade of glutamate-mediated neurotransmission, the amelioration of β-amyloid toxicity, and the modulation of various signaling pathways [49,50,51,52]. Both memantine and KYNA are believed to affect cholinergic neurotransmission through blockade of the α7 subunit of nicotinic receptors. KYNA seems to have a much higher affinity for α7 nicotinic receptors than memantine [5], although some studies have failed to confirm KYNA’s effects on these receptors [6]. Prolonged exposure to β-amyloid peptide increases the number of hippocampal α7 nicotinic receptors [53], and an increased expression correlates positively with the degree of neuropathological changes [54]. Furthermore, β-amyloid binds with a high affinity to nicotinic α7 receptors [55]. Thus, modulating the function of nicotinic α7 receptors may be one of the potential therapeutic actions to prevent β-amyloid peptide-induced cytotoxicity. 

In this context, the memantine-induced stimulation of KYNA production in astrocytes, which is likely followed by enhanced blockade of astrocytic α7 nicotinic receptors, could be a mechanism partly contributing to memantine’s therapeutic effects in dementia. Furthermore, as KYNA alleviates excitotoxicity and displays anti-inflammatory properties, the observed rise in KYNA after memantine administration seems to further boost the neuroprotective effects of this drug.

Considering that higher brain KYNA may impair memory and learning processes mediated via NMDA receptors, the cortical selectivity of memantine seems of special value. As KYNA levels in the hippocampus, pivotal for learning and memory, were not targeted, the major effects of the memantine-mediated cortical increase in KYNA should primarily ameliorate excitotoxic events. Indeed, the combined application of L-KYN and probenecid, which increased the central concentration of KYNA, was demonstrated to improve spatial memory, reduce cellular damage, and decrease reactive gliosis in the CA1 area of rats injected intra-hippocampally with β-amyloid [56].

It was reported that in triple-transgenic AD (3xTg AD) mice, exhibiting both Aβ and tau pathologies, the kynurenine pathway is altered. The baseline expression of the rate-limiting enzymes TDO and IDO was high in the cerebellum compared to the cortex and hippocampus, and it increased with age. At 7 months of age, but not at 12, the expression of TDO was higher in 3xTgAD mice compared to the wild type. In terms of metabolites, hippocampal QUIN increased in an age-dependent way, in contrast to TRP, KYN, and picolinic acid [17]. These data suggest that during the course of AD, the kynurenine pathway is activated, with an ensuing overabundance of neurotoxic QUIN in the hippocampal area. Nevertheless, the levels of KYNA were not studied in this model. Thus, cortical KYNA metabolism in 3xTgAD mice is unknown. However, it is conceivable to assume that an enhanced conversion of KYN to QUIN will deplete the pool of KYN, a substrate for KATs. Consequently, KYNA synthesis should decline. Others, however, have demonstrated that in the whole brain tissue of 29–32-week-old 3xTg AD mice, KYN decreased, while KYNA increased [57]. Considering that the above study did not provide data on the regional distribution of KYNA in the brains of mice, it does not fully oppose the results of Wu et al. [17], and it cannot be excluded that, locally, KYNA levels are reduced.

During the early phases of neurodegeneration and dementia, structural and metabolic dysfunction develops slowly and leads to initially discrete neuronal loss [58,59]. An increasing amount of data implicate astrocytic defects as important factors in brain disorders [60]. Considering the above, our observation on the ability of memantine to increase the levels of astrocyte-derived KYNA may provide another rationale behind the ability of this drug to alleviate the symptoms of cortical pathologies of different origins. 

The potential limitations of our research include studying only one dose of memantine. Furthermore, our experimental design did not involve the assessment of the potential neuroprotective outcomes of increased formation of cortical KYNA. Such research should follow in the future.

## 5. Conclusions

The concept of the early deficit of KYNA which precedes the development of neuronal loss in the brain is supported by significant data [3]. However, during the advanced stages of neurodegeneration, the progressive loss of neurons is accompanied by reactive astrogliosis. Consequently, the ensuing overabundance of glial cells results in higher KYNA production, which may be considered symptomatic and not causative. 

Hence, selective cortical increases in KYNA seem to represent one of the mechanisms contributing to the clinical efficacy of memantine. It is tempting to hypothesize that an early therapeutic intervention based on the combination of memantine with drugs boosting cortical KYNA could provide a more effective option for treating cortical pathologies.

Further investigations should aim to clarify whether prolonged application of memantine is indeed able to exert neuroprotective effects selectively in the cortex, and in a KYNA-dependent manner. The development of selective pharmacological tools allowing for manipulations of the KP within specific brain structures with the use of a region-selective inhibitor of KAT seems vital in this regard. However, substances with such pharmacological profiles are still not available. Research using experimental animal models and clinical studies aiming to verify the potential correlation of the efficacy of memantine with CSF KYNA levels should follow.

## Figures and Tables

**Table 1:** The effects of acute administration of memantine on the ratios between the studied kynurenines.

Brain Structure	Ratio	Control	Memantine
Cortex	TRP/L-KYN	28.28 ± 2.41	29.29 ± 2.68
	TRP/KYNA	927.36 ± 250.71	838.35 ± 168.35
	L-KYN/KYNA	32.80 ± 8.93	28.18 ± 4.48
	3-HK/KYNA	1.79 ± 1.21	1.55 ± 0.4
	L-KYN/3-HK	26.71 ± 19.69	18.88 ± 3.57
	TRP/3-HK	749.31 ± 532.23	563.15 ± 119.93
Striatum	TRP/L-KYN	3.07 ± 0.56	3.03 ± 0.48
	TRP/KYNA	149.40 ± 37.36	123.73 ± 45.27
	L-KYN/KYNA	48.76 ± 12.14	41.74 ± 18.38
	3-HK/KYNA	10.02 ± 3.36	8.66 ± 3.48
	L-KYN/3-HK	5.48 ± 2.77	4.93 ± 1.34
	TRP/3-HK	15.84 ± 4.52	14.64 ± 3.46
Hippocampus	TRP/L-KYN	131.98 ± 100.98	82.62 ± 33.85
	TRP/KYNA	5730.12 ± 631.82	3453.57 ± 768.65
	L-KYN/KYNA	51.58 ± 36.51	48.92 ± 22.79
	3-HK/KYNA	0.18 ± 0.2	0.11 ± 0.03
	L-KYN/3-HK	379.65 ± 286.47	444.32 ± 344.32
	TRP/3-HK	31,612.86 ± 6224.3	30,379.05 ± 4953.80
Cerebellum	TRP/L-KYN	2.76 ± 0.27	3.02 ± 0.41
	TRP/KYNA	2447.12 ± 1695.8	2674.72 ± 1731.6
	L-KYN/KYNA	878.78 ± 551.86	932.41 ± 684.18
	3-HK/KYNA	2.94 ± 2.06	2.76 ± 1.88
	L-KYN/3-HK	309.38 ± 50.33	337.17 ± 62.25
	TRP/3-HK	846.61 ± 92.04	979.53 ± 241.18

Memantine was given at a dose of 20 mg/kg i.p. The ratios between kynurenines were estimated separately for each animal, and subsequently, mean values were established. TRP—tryptophan, KYNA—kynurenic acid, L-KYN—L-kynurenine, 3-HK—3-hydroxykynurenine.

**Table 2:** The effects of 5-day administration of memantine on the ratios between the studied kynurenines.

Brain Structure	Ratio	Control	Memantine
Cortex	TRP/L-KYN	22.75 ± 3.32	21.77 ± 5.32
	TRP/KYNA	884.59 ± 215.72	538.89 ± 133.31 ***
	L-KYN/KYNA	38.91 ± 7.81	25.26 ± 6.02 ***
	3-HK/KYNA	1.81 ± 7.81	1.31 ± 0.45 *
	L-KYN/3-HK	22.16 ± 4.18	25.97 ± 26.97
	TRP/3-HK	505.73 ± 127.97	550.86 ± 571.44
Striatum	TRP/L-KYN	3.60 ± 1.2	3.95 ± 0.99
	TRP/KYNA	299.95 ± 408.26	181.32 ± 68.63
	L-KYN/KYNA	93.86 ± 138.85	49.90 ± 24.95
	3-HK/KYNA	22.16 ± 30.61	11.39 ± 5.33
	L-KYN/3-HK	4.51 ± 1.94	4.44 ± 1.4
	TRP/3-HK	15.31 ± 5.62	17.15 ± 5.64
Hippocampus	TRP/L-KYN	62.16 ± 16.1	63.61 ± 13.45
	TRP/KYNA	3802.30 ± 1049.0	3931.92 ± 574.18
	L-KYN/KYNA	65.08 ± 26.44	65.13 ± 20.93
	3-HK/KYNA	0.12 ± 0.04	0.12 ± 0.04
	L-KYN/3-HK	543.07 ± 107.78	609.24 ± 32.34
	TRP/3-HK	32,729.5 ± 7261.1	36,714.22 ± 17,194.0
Cerebellum	TRP/L-KYN	3.61 ± 0.36	3.95 ± 0.73
	TRP/KYNA	30,569.45 ± 2183.7	6036.42 ± 7106.3
	L-KYN/KYNA	829.24 ± 550.91	1608.47 ± 1765.0
	3-HK/KYNA	2.73 ± 1.68	4.90 ± 4.39
	L-KYN/3-HK	309.89 ± 73.12	318.28 ± 84.6
	TRP/3-HK	1111.99 ± 251.04	1260.31 ± 417.52

Memantine was given at a dose of 20 mg/kg i.p. The ratios between kynurenines were estimated separately for each animal, and subsequently, mean values were established. Statistical significance * *p* < 0.05 vs. control; *** *p* < 0.001 vs. control. TRP—tryptophan, KYNA—kynurenic acid, L-KYN—L-kynurenine, 3-HK—3-hydroxykynurenine.

## Data Availability

The data that support the findings of this study are available from the corresponding author upon request.
